# Identification and evaluation of a new entomopathogenic fungal strain against *Riptortus pedestris* (Hemiptera: Alydidae) and its two egg parasitoids

**DOI:** 10.1371/journal.pone.0195848

**Published:** 2018-04-17

**Authors:** Naresh Dangi, Un Taek Lim

**Affiliations:** 1 Entomology Division, Nepal Agricultural Research Council, Khumaltar, Lalitpur, Nepal; 2 Department of Bioresource Sciences, Andong National University, Andong, Republic of Korea; 3 Institute of Agricultural Science and Technology, Andong National University, Andong, Republic of Korea; Montana State University Bozeman, UNITED STATES

## Abstract

A strain (ARP14) of the entomopathogenic fungus *Beauveria bassiana* (Balsamo) Vuillemin was isolated from field-collected *Riptortus pedestris* (Fabricius) (Hemiptera: Alydidae). The lethal median concentration of the ARP14 strain was compared with that of a commercialized strain (GHA) of the same fungus against *R*. *pedestris* and its two egg parasitoids, *Ooencyrtus nezarae* Ishii (Hymenoptera: Encyrtidae) and *Gryon japonicum* (Ashmead) (Hymenoptera: Platygastridae). Mortality and mycosis rates were evaluated after exposure to five concentrations of the fungus, i.e., 1×10^9^, 1×10^8^, 1×10^7^, 1×10^6^, and 1×10^5^ conidia/mL, using a glass scintillation vial as an exposure arena in 25.0 ± 0.5°C and 93.7 ± 2.9% RH. The lethal median concentrations (LC_50_) for 2^nd^ and 4^th^ instar nymphs, and adults of *R*. *pedestris* were not significantly different between the two strains of *B*. *bassiana*. However, the mycosis rate of ARP14 was 1.3 and 1.8 times higher than that of the GHA strain in 4^th^ instar nymphs and adult females of *R*. *pedestris*, respectively, at the 1×10^8^ conidia/mL concentration. More interestingly, the mycosis rates at 1×10^8^ conidia/mL concentration in the parasitoids *G*. *japonicum* and *O*. *nezarae* were much lower in the ARP14 strain (15.0 and 0%) than in the GHA strain (73.3 and 66.0%), respectively, suggesting that the *B*. *bassiana* strain ARP14 is less virulent to these parasitoids than the commercially available strain. Our results suggest that *B*. *bassiana* ARP14 may be a potential new biopesticide against *R*. *pedestris* with fewer negative effects on beneficial parasitoids than currently available options.

## Introduction

Pod sucking bugs, including *Riptortus pedestris* (F.) (Hemiptera: Alydidae), *Halyomorpha halys* (Stål) (Hemiptera: Pentatomidae), and *Nezara antennata* Scott (Hemiptera: Pentatomidae), are major pests of soybean by reducing seed quality and yield [1, 2]. Among these bugs, *R*. *pedestris* is a major soybean pest in Korea and Japan [3, 4]. While conventional management practices against *R*. *pedestris* include two or three applications of broad spectrum insecticides during the soybean reproductive phase [5, 6], chemical insecticides are neither efficient nor compatible with biological control agents. The conservation of parasitoids using less toxic insecticides or mycoinsecticides has potential to reduce problems from broad spectrum insecticides and improve the sustainable management of *R*. *pedestris* in soybean.

*Beauveria bassiana* (Balsamo) Vuillemin is a widely distributed, diverse, and well-studied entomopathogenic fungus (EPF) that infects insect pests in many orders [7] and is a promising bio-control agent for managing a number of insect pests [8,9]. Some strains such as IRS49, IPP233, and ARSEF210087 of *B*. *bassiana* have shown high levels of mortality and infection in non-target organisms [10] as well as the targeted pests [11–18], while other *B*. *bassiana* strains (i.e., GHA, JW-1 and ARSEF 3113) are relatively harmless to many non-target organisms [10,19,20]. The virulence and mycosis rates caused by *B*. *bassiana* varies with host insect species and enzymatic traits of particular fungi or strains [21].

Several *B*. *bassiana* isolates have been assessed and found to be promising biological control agents for hemipteran bugs [22]. In recent years, several studies have assessed the efficacy of *B*. *bassiana* against different hemipteran bugs. The GHA strain of *B*. *bassiana* was found to infect 2^nd^ instars of *H*. *halys* quite well [23]. However, that strain of *B*. *bassiana* has never been isolated from *R*. *pedestris*. In this study, we report a new strain of *B*. *bassiana*, designated ARP14, which was isolated from a *R*. *pedestris* cadaver collected in Songcheon, Andong, Republic of Korea in 2014. As EPF are usually detrimental to natural enemies [24], we assessed the virulence of *B*. *bassiana* ARP14 to two egg parasitoids of *R*. *pedestris* as well as different life stages of *R*. *pedestris*. The parasitoids included the gregarious *Ooencyrtus nezarae* Ishii (Hymenoptera: Encyrtidae) and the solitary *Gryon japonicum* (Ashmead) (Hymenoptera: Platygastridae). The specific objectives of this study were (1) to identify the EPF isolated from *R*. *pedestris* and (2) to compare the relative virulence of *B*. *bassiana* ARP14 with the commercially available *B*. *bassiana* GHA strain against 2^nd^ instar nymphs, 4^th^ instar nymphs, and adult females of *R*. *pedestris*, as well as its two egg parasitoids. Information on the virulence and epizootic conditions of *B*. *bassiana* on this pest and its parasitoids will be crucial for developing a new strain of EPF to use as control agents of *R*. *pedestris* in soybean.

## Materials and methods

### Isolation and mass production of pathogen

An adult *R*. *pedestris* infected with *B*. *bassiana* was collected from a soybean field in Songcheon, Andong, Republic of Korea in 2014. The infected insect has been preserved in sterilized falcon tube in a freezer. The fungus was isolated and cultured in Sabouraud Dextrose Agar (SDA) media for 14 d. After isolating the fungus from the host, a single colony was removed and cultured, adding the antibiotics streptomycin (100 mg/L), cycloheximide (70 mg/L), and tetracycline (50 mg/L) to suppress other organisms and produce a pure culture of the *B*. *bassiana* isolate after 2 times of plating [25]. The purified fungal culture was replated using the loop streak dilution method. A single colony of the fungus was isolated and transferred after 72 h and then grown for 14 d.

### Insects

*Riptortus pedestris* and its egg parasitoids, *O*. *nezarae* and *G*. *japonicum*, were reared and maintained in the laboratory following Kim and Lim [26]. Adults of *R*. *pedestris* were reared on dry soybean (*Glycine max* [L.] Merr. var. Daewon) seeds and cotton soaked with 2% vitamin C water solution in acrylic cages (40 L× 40 W × 40 H cm). Eggs were collected from gauze (the oviposition substrate) that was hung in the corners of the cage. *Riptortus pedestris* nymphs were reared in a separate cage with potted kidney bean (*Phaseolus vulgaris* L.) plants, dry soybean seeds, and cotton soaked with a 2% vitamin C water solution. The egg parasitoids of *R*. *pedestris* were reared on non-viable host eggs (refrigerated for 25–30 d) according to Alim and Lim [27] in a centrifugal tube (50 mL) with a streak of honey on the wall. Mated females (3 d old) of the egg parasitoids were released for 24 h in the centrifugal tubes containing the non-viable host eggs, and the parasitized eggs were incubated at 27.2 ± 1.0°C, 41.7 ± 8.7% RH, and a 16:8 h L: D photoperiod. Emerged parasitoid adults were collected in the centrifugal tube for bioassays and further rearing.

### Morphological and molecular identification of *B*. *bassiana* strains

The morphology of the fungal pathogen’s synnema was studied under scanning electron microscopy (MIRA3, Tescan Orsay Holding, Brno-Kohoutovice, Czech Republic) according to the taxonomic description of Rehner et al. [8]. DNA of each of the two fungal strains was extracted following the methodology described by Chi et al. [28]. About 20 mg of fungal mycelia (2–3 d old grown in SDA media) was harvested with a sterilized dissection blade and put into an Eppendorf tube (1.5 mL) containing 300 μL of extraction buffer [1 M KCl.100 mM Tris-HCl (pH 8.0), 10 mM EDTA]. Mycelia tissues were thoroughly ground using a pestle, followed by centrifugation at 11,000×g for 1 min. The supernatant was transferred to a sterile Eppendorf tube, and isopropanol (200 μL) was added. The tube was well mixed before additional centrifugation at 11,000×g for 10 min. The supernatant was discarded, 300 μL of ethanol was added, and the tube was gently inverted to wash the pellet three times followed by a final centrifugation at 11,000×g for 1 min. The supernatant was discarded, and the Eppendorf tube was left open at room temperature to allow excess ethanol to evaporate. After 10 min, the DNA pellet was gently dissolved in 50 μL of 1×TE buffer by tapping the tube. The ITS-rDNA region of the collected DNA sample was amplified using primer pairs- ITS1 (5'-TCC GTA GGT GAA CCT GCG G-3') and ITS4 (5'-TCC TCC GCT TAT TGA TAT GC-3') [29] in a SimpliAmp Thermal Cycler (Life Technologies Holding Pte Ltd, Singapore). The PCR extraction was done by preheating the sample at 95°C for 5 min, followed by 35 incubation cycles at 94°C for 45 sec, 55°C for 30 sec, and 72°C for 45 sec followed by a final extension at 72°C for 5 min. The PCR product was purified using a PCR purification kit (Biofact Co., Ltd., Daejeon, Republic of Korea) and sequenced using ABI PRISM 3730XL analyzer by Macrogen Korea (Seoul, Republic of Korea).

The nucleotide sequence of the APR14 (Accession No. MG952537.1) strain was compared with that of the other *B*. *bassiana* strain using a Blast search of sequences from the NCBI Genbank database. The nucleotide sequences most similar to ARP14 and that of the fungal species most closely related to *B*. *bassiana*, *Isaria* spp., and *Metarhizium* spp. were downloaded from the Genbank, and phylogenetic analysis of these taxa was conducted using MEGA7 software (Biodesign Institute, Tempe, Arizona).

### Preparation of conidia suspension

*Beauveria bassiana* ARP14 and *B bassiana* GHA (Botanigard^®^ ES, Laverlam International Cooperation, Parkmont, Butt, MT) were grown under dark conditions at 25.0 ± 1.0°C and 50.0 ± 10.0% RH for 14 d. Conidia suspensions of the two strains were prepared by scraping the fungal culture into a 20 mL liquid scintillation (LS) vial (240804, Wheaton, Millville, NJ) containing autoclaved Triton X-100 (0.1%) solution (Duksan Pure Chemicals Co. Ltd., Ansan, Republic of Korea). The suspension was agitated for 2–5 min using Vortex mixer (KMC-1300V, Vision Scientific Co. Ltd, Daejeon, Republic of Korea) to separate the conidia clumps. Conidial concentrations of the suspension were measured using Neubauer hemocytometer (Marienfeld-Superior, Paul Marienfeld GmbH and Co. KG, Lauda-Königshofen, Germany) under a 40× microscope [25]. Based on the count, we set the suspension to the concentration of 1×10^9^ conidia/mL, and prepared other solutions in different concentration by serial dilution: 1×10^9^, 1×10^8^, 1×10^7^, 1×10^6^, and 1×10^5^ conidia/mL.

### *Beauveria bassiana* toxicity in a glass vial assay

Three different life stages of *R*. *pedestris* (<24 h old 2^nd^ instar nymphs, <36 h old 4^th^ instar nymphs, and <48 h old adult females) and adult females of its two egg parasitoids, *G*. *japonicum* and *O*. *nezarae* (both 5–7 d old), were tested at five different concentrations (1×10^9^, 1×10^8^, 1×10^7^, 1×10^6^, 1×10^5^ conidia/mL) of ARP14 and GHA, using 0.1% Triton X-100 ddH_2_O as a control. The 20 mL LS vial was used for the bioassay, and we coated the inside of each vial with 100 μL of the test solution for each concentration and air dried in room temperature. For each replicate of each species or stage, five insects were exposed for 12 h in the fungus-coated vials. Exposed insects were then transferred to clean 2 mL Eppendorf tubes with a small hole in lid after 12 h of exposure and kept in desiccators (4202–0000, Bel-Art Products, Pequannck, NJ) at 25.0 ± 0.5°C and 93.7 ± 2.9% RH inside a growth chamber (DS-50CPL, Dasol Scientific Co., Ltd, Suwon, Republic of Korea) to determine the fungal mycosis development rate over a 14 d period following exposure. RH inside desiccators was maintained using saturated Potassium Sulfate (K_2_SO_4_) solution [30]. Water and food were not provided for the insects to remove the compounding effects on pathogens. Temperature and RH during the experiment was measured using a data logger (H8-003-02, Onset Computer Corporation Bourne, MA) inside the desiccators. Mortality of insects was observed at 12 h intervals from exposure until death. Insect was categorized as death when there was no movement during three times touch with a camel brush under stereoscopic microscope. Insects categorized as mycosis with *B*. *bassiana* when fungus mycelia were visible on insects’ integument through a stereoscopic microscope.

### Statistical analysis

Mortality from the various concentrations of the ARP14 and GHA strains were subjected to log-probit regression analysis to calculated lethal median time (LT_50_), based on observations every 12 h after exposure. The mortality data from trials with each fungal strain and concentration for both *R*. *pedestris* and its parasitoids were also used to calculate the lethal median concentration (LC_50_) [31]. Significant differences among treatments were determined based on the 95% confidence interval (CI). The toxicity index at different concentration levels was calculated by dividing the LT_50_ of each control with that of the treatment [32]. The fungal mycosis development rates of the ARP14 and GHA strains were analyzed with normal approximation of the chi-square test, and Tukey type multiple comparison test was followed (α = 0.05) [33]. Comparison of data for the mortality and fungal mycosis rates between ARP14 and GHA strains at each concentration and insect stage or species was conducted using two proportion Z-tests [33].

## Results

### Morphology and phylogenetics of *Beauveria bassiana* ARP14

**Fig 1:**
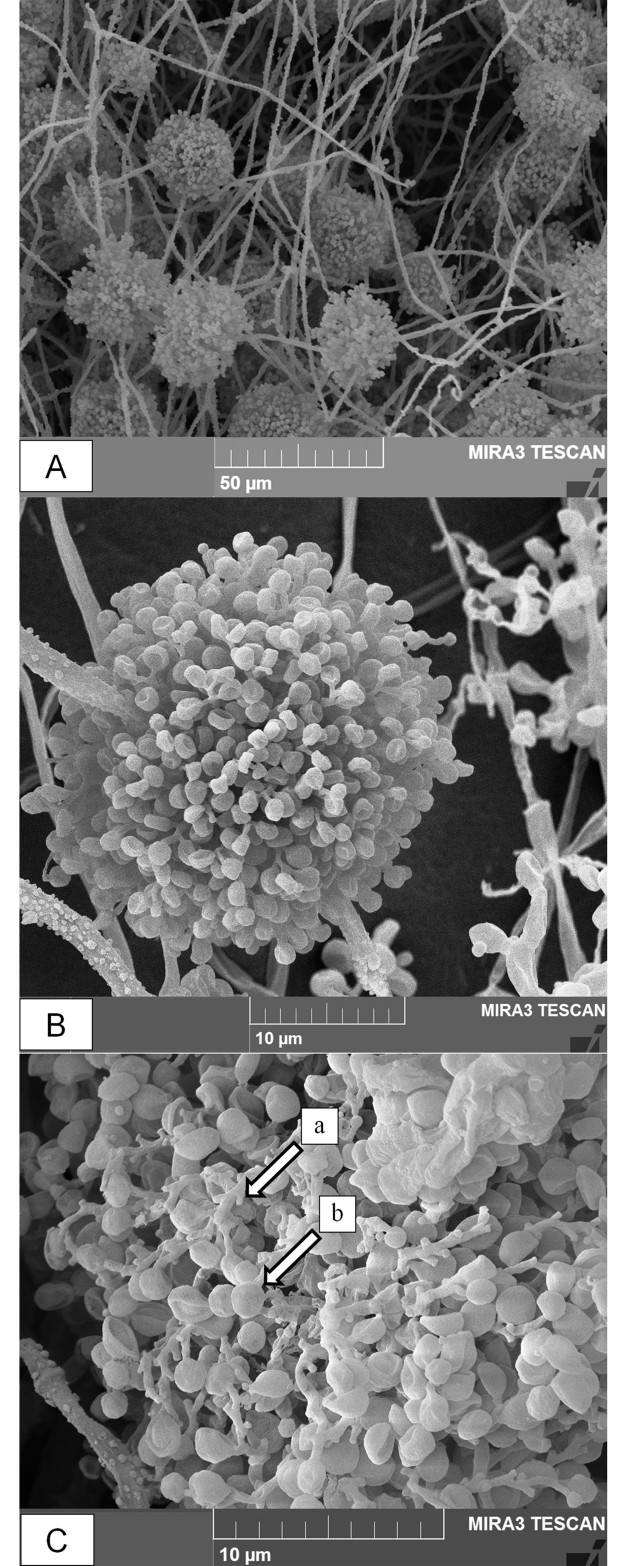
Scanning electron micrographs of *B*. *bassiana* ARP14 on the cadaver of adult *Riptortus pedestris* (F.) (Hemiptera: Alydidae). (A) Group of clustered conidigenous cells on insect cadaver magnification: 1.49 K×), (B) Short globose shaped cluster of conidigenous cells (magnification: 7.5 K×), and (C) Conidia shape and rachis structure (magnification: 10.1 K×). Arrows indicating (a) the denticulate rachis elongated in a long zig-zag extension (b) conidia having globose shaped terminated in a narrow apical extension of rachis.

**Fig 2:**
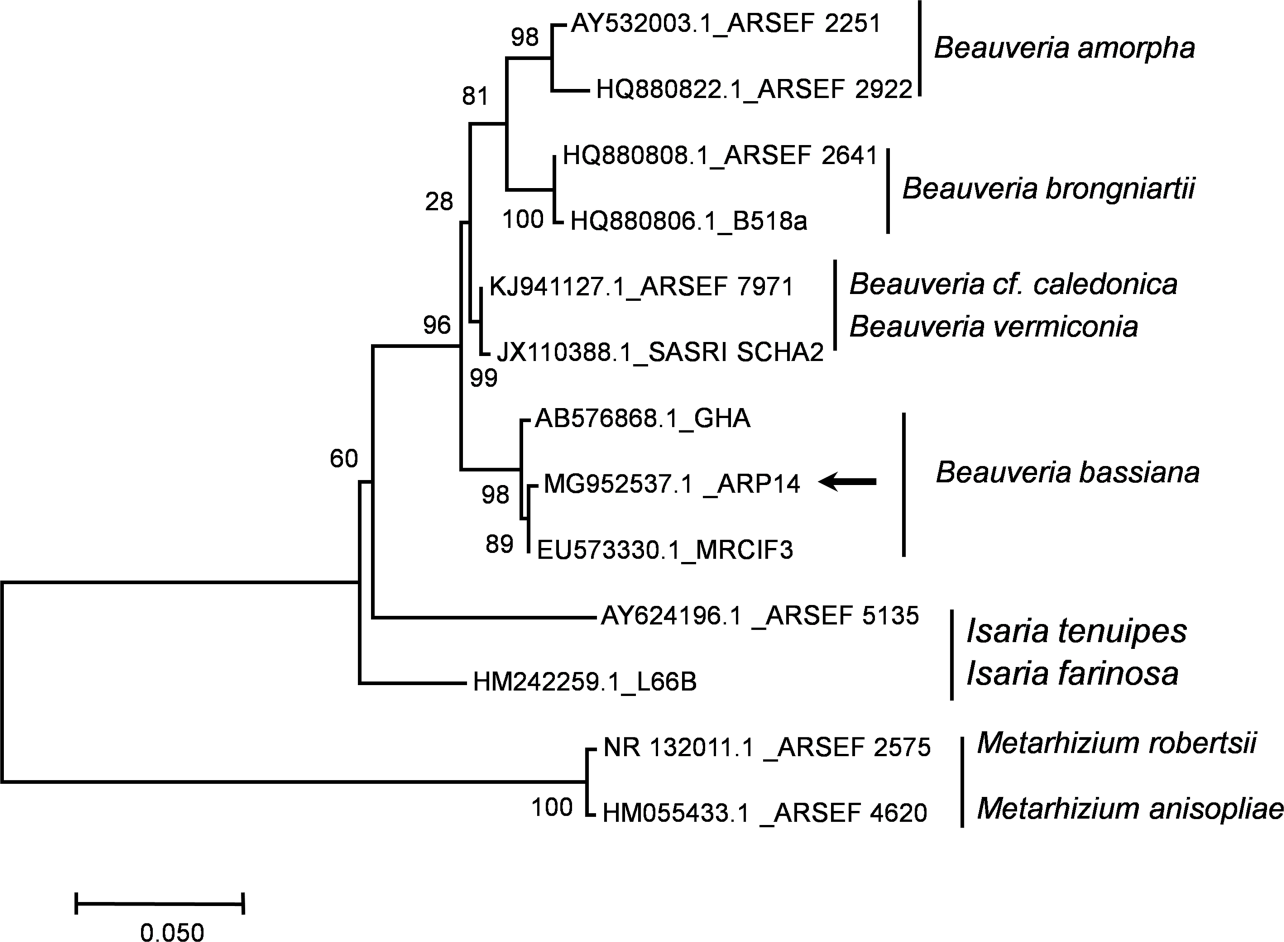
Molecular phylogenetic analysis of *Beauveria bassiana* ARP14 with similar strain and other genera based on nucleotide sequence and constructed by maximum likelihood method. The analysis involved 13 nucleotide sequences. All positions containing gaps and missing data were eliminated. A total of 438 positions were included in the final dataset. Evolutionary analyses were conducted in MEGA7.

The strain spore ball composed of short-globose shaped clusters of conidiogenous cells (Fig 1B), and conidia terminated in a rachis narrow apical extension (Fig 1C-a). The zig-zag extension of elongated rachis formed globose to subglobose shaped conidia. The intraspecies divergence rate was 0.004±0.002 among the 3 species of *B*. *bassiana*. Similarly, interspecies divergence rate of *B*. *bassiana* ARP14 with *B*. *vermiconia*, *B*. *amorpha*, and *B*. *brongniartii* was 0.018±0.008, 0.031±0.012, and 0.032±0.013, respectively. Thus, this strain ARP14 is designated as *B*. *bassiana* type clade (Fig 2).

### *Beauveria bassiana* toxicity to *R*. *pedestris* in a glass-vial assay

**Fig 3:**
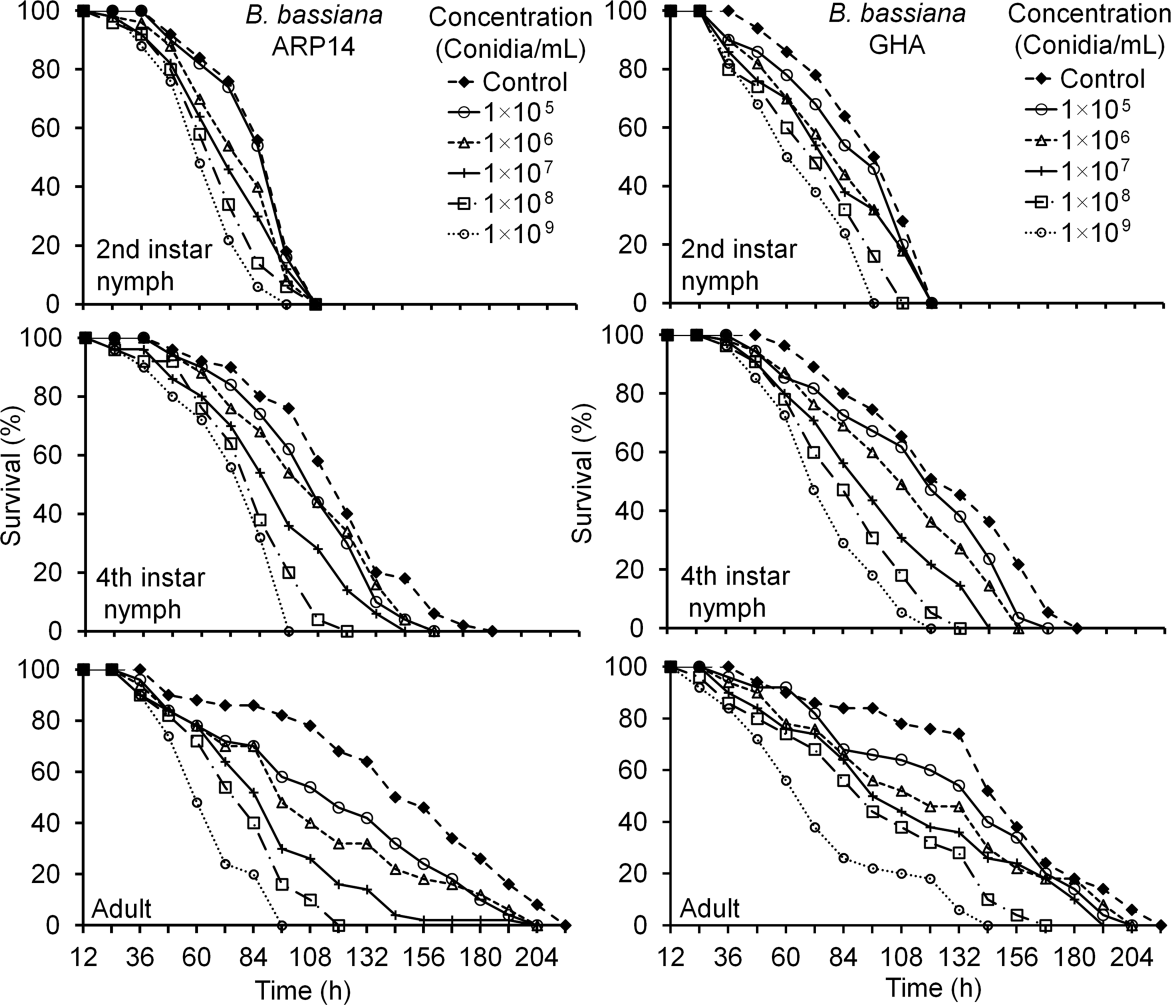
Survivorship of different life stages of *Riptortus pedestris* exposed to different concentrations of *Beauveria bassiana* of the ARP14 or GHA strains.

*Riptortus pedestris* nymphs and adults died faster at higher conidial concentrations (e.g., 1×10^9^ and 1×10^8^ conidia/mL) for both *B*. *bassiana* strains tested (ARP14 and GHA) (Table 1; Fig 3) than at lower conidial concentrations. The LC_50_ values for bug either nymphs or adults were not significantly different between the two strains (ARP14 and GHA) (Table 2). The mortality rates of 2^nd^ instar nymphs at either 48 (*Z* = 0.71, *P* = 0.476) or 72 h (*Z* = 1.42, *P* = 0.155) were not significantly different between the ARP14 and GHA strains at the 1×10^8^ conidia/mL concentration. The 2^nd^ instar nymphs of *R*. *pedestris* showed 100% mortality 108 h after exposure to 1×10^8^ conidia/mL concentration of either *B*. *bassiana* ARP14 or GHA. Similarly, the mortality rates of 4^th^ instar nymphs at 48 (*Z* = 0.20, *P* = 0.842), 72 (*Z* = 0.42, *P* = 0.673), or 96 h (*Z* = 1.28, *P* = 0.201) were not significantly different between the ARP14 and GHA strains. For 4^th^ instars of *R*. *pedestris*, 100% mortality was observed for strain ARP14 at 120 h post exposure, while for strain GHA all nymphs were not dead until 132 h after exposure, in both cases at the 1×10^8^ conidia/mL concentration. The same general effect of strain, over time for different concentrations, was observed for adult females of *R*. *pedestris* as for 4^th^ instar nymphs, with no difference between strains at 48 (*Z* = 0.25, *P* = 0.799) and 72 h (*Z* = 1.44, *P* = 0.151). Mortality was, however, significantly different between strains at 96 h (*Z* = 3.06, *P* = 0.002). Adult mortality reached 100% at 120 and 168 h after the exposure to 1×10^8^ conidia/mL concentration for the ARP14 and GHA strains, respectively (Fig 3).

**Table 1:** Lethal median time (LT_50_) for different life stages of *Riptortus pedestris* after a 12 h exposure to conidia of *Beauveria bassiana* ARP14 and GHA strains in a glass-vial assay.

Strain	Life stage	Concentration (conidia/mL)	LT_50_(h)	95% C.I.^†^ (Lower—Upper)		Toxicity index*		χ^2^ (df)
ARP14	2^nd^ instar nymphs (n = 50)	1×10^9^	56.3a	53.3–59.2	1.4		7.0 (6)	
		1×10^8^	59.8a	52.3–67.3	1.3		21.1 (7)	
		1×10^7^	65.2a	59.1–71.5	1.2		12.5 (7)	
		1×10^6^	69.2ab	57.5–82.4	1.1		41.9 (7)	
		1×10^5^	77.9b	71.0–86.3	1.0		22.5 (7)	
		Control	78.9b	71.6–87.4	1.0		21.7 (7)	
	4^th^ instar nymphs (n = 50)	1×10^9^	66.6a	55.3–83.9	1.6		26.4 (6)	
		1×10^8^	71.2a	56.8–87.6	1.5		65.3 (8)	
		1×10^7^	79.8ab	69.2–91.0	1.3		41.6 (10)	
		1×10^6^	94.6bc	90.3–99.0	1.1		15.2 (11)	
		1×10^5^	96.5bc	89.8–103.5	1.1		23.3 (11)	
		Control	106.1c	99.0–113.2	1.0		29.4 (13)	
	Adult (n = 50)	1×10^9^	58.1a	54.8–61.3	2.3		6.7 (6)	
		1×10^8^	69.4b	63.3–75.5	1.9		13.9 (8)	
		1×10^7^	78.1b	73.8–82.3	1.7		13.8 (15)	
		1×10^6^	93.8c	88.3–99.3	1.4		11.2 (15)	
		1×10^5^	101.7c	96.0–107.6	1.3		22.3 (15)	
		Control	133.0d	120.5–147.4	1.0		51.8 (16)	
GHA	2^nd^ instar nymphs (n = 50)	1×10^9^	58.1a	51.5–65.1	1.5		11.8 (6)	
		1×10^8^	62.9ab	55.3–71.0	1.4		16.2 (7)	
		1×10^7^	70.1ab	63.1–77.6	1.3		14.5 (8)	
		1×10^6^	73.0b	68.7–77.5	1.2		13.1 (8)	
		1×10^5^	79.6bc	69.8–91.8	1.1		25.3 (8)	
		Control	87.8c	80.9–96.1	1.0		17.5 (8)	
	4t^h^ instar nymphs (n = 55)	1×10^9^	69.2a	65.9–72.5	1.7		5.5 (8)	
		1×10^8^	77.2b	73.6–80.8	1.5		9.6 (9)	
		1×10^7^	86.1c	82.0–90.4	1.4		8.2 (10)	
		1×10^6^	98.8d	94.1–103.7	1.2		14.5 (11)	
		1×10^5^	105.8de	97.3–115.2	1.1		30.9 (12)	
		Control	117.3e	112.8–122.0	1.0		18.2 (13)	
	Adult (n = 50)	1×10^9^	62.0a	57.4–66.5	2.1		9.1 (10)	
		1×10^8^	82.2b	73.8–90.9	1.6		23.6 (12)	
		1×10^7^	95.5bc	89.5–101.5	1.4		14.8 (14)	
		1×10^6^	103.4cd	97.4–109.4	1.3		16.4 (15)	
		1×10^5^	115.6de	105.9–125.8	1.1		33.4 (15)	
		Control	132.4e	120.0–146.3	1.0		61.0 (16)	

^†^Confidence interval.

*LT_50_ of control/ LT_50_ of treatment (Sun 1950).

LT_50_ value followed by different small letters is significantly different among the concentration in 95% C.I.

**Table 2:** Lethal median concentration (LC_50_) of *Beauveria bassiana* ARP14 and GHA strains against *Riptortus pedestris* exposed for 12 h in a glass-vial assay (n = 300).

Insect stage	Strain	Slope (± SE)	LC_50_	95% CI^†^ (Lower- Upper)	χ^2^(df)	
LC_50_ 72 h after exposure						
2^nd^ instar nymphs	ARP14	0.3 ± 0.1	4.6×10^6^	1.2×10^6^−1.4×10^7^	0.72 (3)	
	GHA	0.2 ± 0.1	2.9×10^7^	3.4×10^6^−9.1×10^8^	0.24 (3)	
LC_50_ 96 h after exposure						
4^th^ instar nymphs	ARP14	0.5 ± 0.1	9.7×10^5^	1.5×10^4^–6.6×10^6^	6.6 (3)
	GHA	0.4 ± 0.1	1.4×10^6^	3.8×10^5^−3.7×10^6^	1.0 (3)
Adult	ARP14	0.5 ± 0.1	5.1×10^5^	1.5×10^5^–1.2×10^6^	4.7 (3)
	GHA	0.3 ± 0.1	5.6×10^6^	1.0×10^6^−2.4×10^7^	2.1 (3)

^†^Confidence interval.

The LT_50_ values of both strains, at concentrations higher than 1×10^6^ conidia/mL, were all lower than that of the buffer control in all the life stages tested (Table 1). The toxicity index presented in Table 1 illustrates the different survivorship among the concentrations of the two strains. Compared to the buffer control, the toxicity index at 1×10^8^ conidia/mL was 1.3, 1.5, and 1.9 times higher in 2^nd^ instar nymphs, 4^th^ instar nymphs, and adult females of *R*. *pedestris* exposed in *B*. *bassiana* ARP14 strain whereas it was 1.4, 1.5, and 1.6 times higher for the *B*. *bassiana* GHA strain, respectively.

### *Beauveria bassiana* toxicity to *R*. *pedestris* egg parasitoids in a glass-vial assay

**Fig 4:**
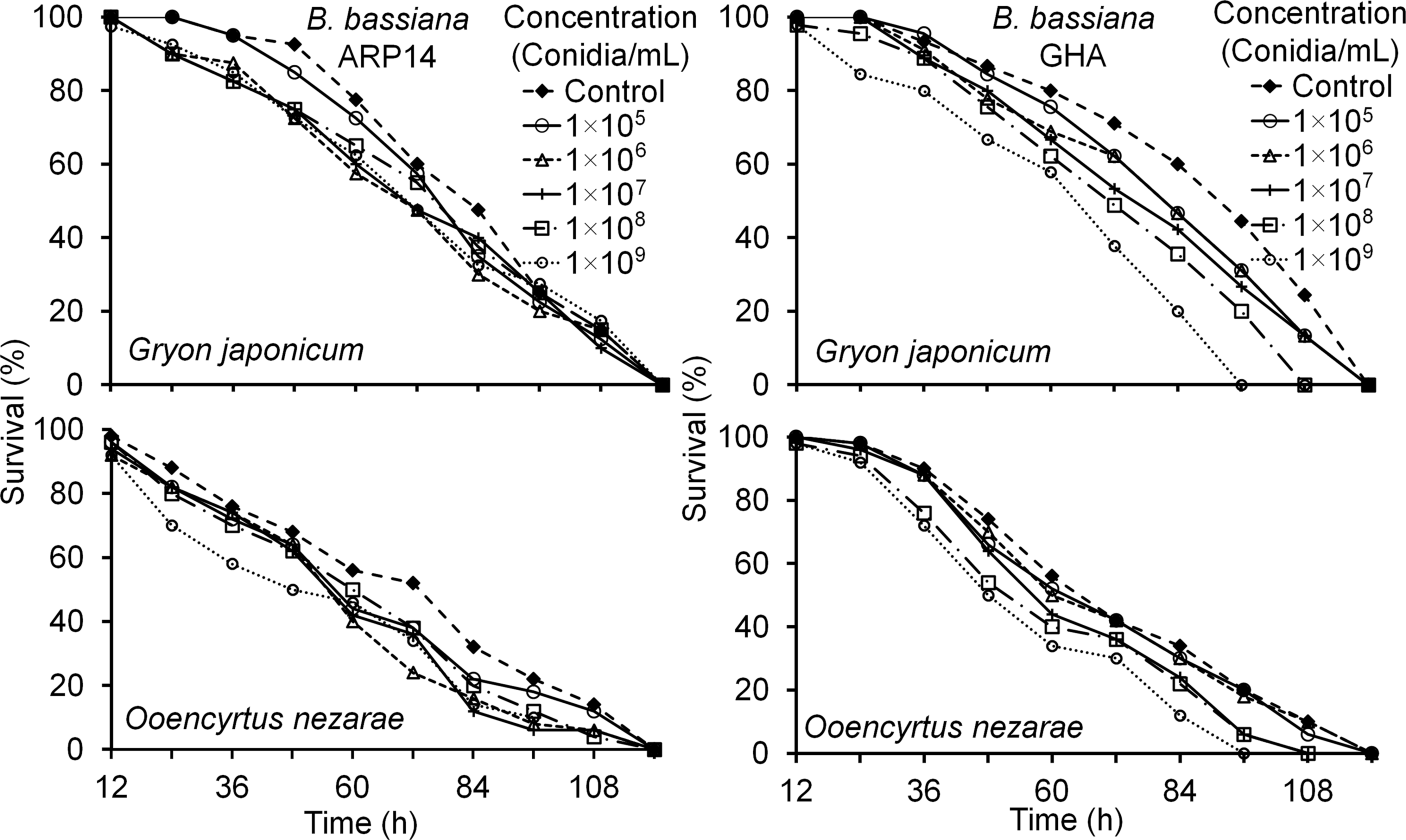
Survivorship of different egg parasitoids of *Riptortus pedestris* exposed to different concentrations of *Beauveria bassiana* of the ARP14 or GHA strains.

The LT_50_ values for both parasitoids of the *B*. *bassiana* ARP14 strain were not significantly different from the control for any of the five conidial concentrations except for mortality of *O*. *nezarae* at 1×10^9^ conidia/mL (Table 3). However, for the GHA strain, parasitoid mortality significantly higher at 1×10^9^ conidia/mL in both species and at 1×10^8^ conidia/mL for *O*. *nezarae*, only compared to the controls (Table 3). The mortality of *G*. *japonicum* at 48 (*Z* = 0.25, *P* = 0.806) and 72 h (*Z* = 1.02, *P* = 0.309) and that of *O*. *nezarae* at 48 (*Z* = 0.81, *P* = 0.417) and 72 h (*Z* = 0.21, *P* = 0.836) were not significantly different between APR14 and GHA strain at 1×10^8^ conidia/mL concentration. In both parasitoids, *G*. *japonicum* and *O*. *nezarae*, 100% mortality occurred at 120 and 108 h after exposure to 1×10^8^ conidia/mL concentration of *B*. *bassiana* ARP14 and GHA, respectively. Nevertheless, in control, 100% mortality occurred 120 h after exposure in both strains (Fig 4).

**Table 3:** Lethal median time (LT_50_) for two egg parasitoids (*Gryon japonicum* and *Ooencyrtus nezarae*) of *Riptortus pedestris* after a 12 h exposure in a glass-vial coated with conidia of *Beauveria bassiana* strains ARP14 or GHA.

Strain	Test insect	Concentration (Conidia/mL)	LT_50_ (h)	95% C.I.^†^ (Lower—Upper)	Toxicity index*	χ^2^ (df)
ARP14	*Gryon japonicum* (n = 40)	1×10^9^	63.5a	53.6–74.5	1.2	18.8 (8)
		1×10^8^	65.1a	56.1–75.0	1.2	16.1 (8)
		1×10^7^	63.2a	54.7–72.2	1.2	15.2 (8)
		1×10^6^	62.2a	57.2–67.2	1.2	12.0 (8)
		1×10^5^	71.8a	67.6–76.1	1.1	5.9 (8)
		Control	76.0a	71.8–80.0	1.0	10.2 (8)
	*Ooencyrtus nezarae* (n = 50)	1×10^9^	41.4a	33.1–49.5	1.4	19.3 (8)
		1×10^8^	49.3ab	40.9–57.6	1.2	20.1 (8)
		1×10^7^	47.4ab	38.2–56.4	1.2	26.0 (8)
		1×10^6^	46.1ab	37.0–55.0	1.3	25.5 (8)
		1×10^5^	51.1ab	46.4–55.7	1.2	12.6 (8)
		Control	59.1b	50.7–68.1	1.0	17.1 (8)
GHA	*Gryon japonicum* (n = 45)	1×10^9^	54.3a	42.7–68.9	1.5	21.1 (6)
		1×10^8^	64.2ab	47.0–88.1	1.3	51.0 (7)
		1×10^7^	70.0ab	65.6–74.5	1.2	10.8 (8)
		1×10^6^	72.5ab	65.3–80.3	1.1	14.7 (8)
		1×10^5^	75.3b	71.1–79.7	1.1	10.9 (8)
		Control	82.2b	73.1–93.8	1.0	20.6 (8)
	*Ooencyrtus nezarae* (n = 50)	1×10^9^	47.6a	40.7–54.4	1.3	11.7 (6)
		1×10^8^	51.4ab	43.8–58.8	1.2	16.1 (7)
		1×10^7^	56.7abc	53.0–60.3	1.1	8.8 (7)
		1×10^6^	61.7bc	57.7–65.7	1.0	5.6 (8)
		1×10^5^	61.2bc	57.2–65.1	1.1	7.8 (8)
		Control	64.0c	60.0–68.0	1.0	6.4 (8)

^†^Confidence interval.

*LT_50_ of control/ LT_50_ of treatment (Sun 1950).

LT_50_ value followed by different small letters is significantly different among the concentration in 95% C.I.

The toxicity index for *G*. *japonicum* and *O*. *nezarae* was 1.2 in each species at 1×10^8^ conidia/mL exposed to the ARP14. Similarly, this index was 1.3 and 1.2 for the GHA strain at 1×10^8^ conidia/mL for *G*. *japonicum* and *O*. *nezarae*, respectively.

### Mycosis rates for *Beauveria bassiana* strains ARP14 and GHA

**Fig 5:**
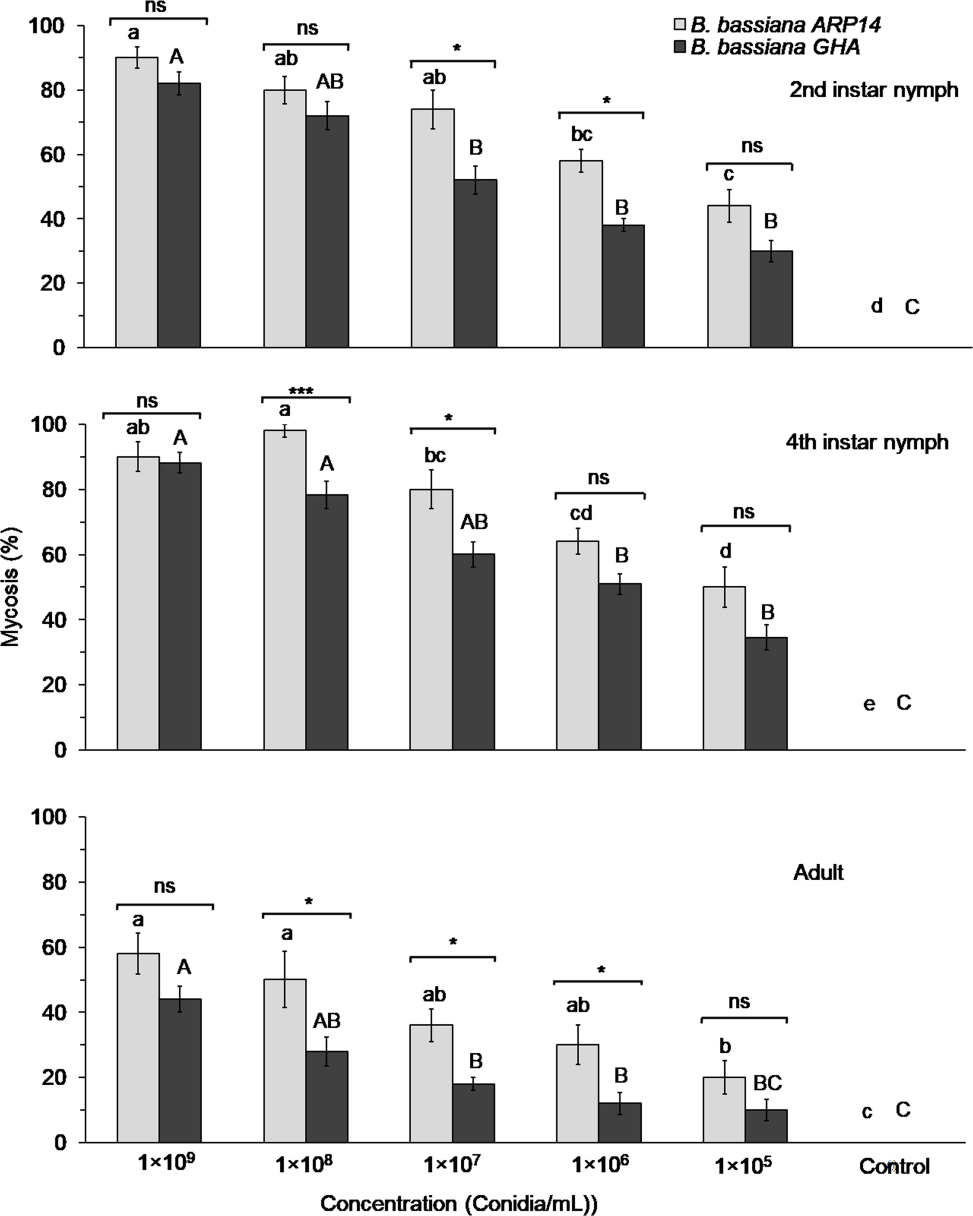
Mycosis rate of *Beauveria bassiana* ARP14 and GHA strains on 2^nd^ instar nymph, 4^th^ instar nymph and adult stage of *Riptortus pedestris* exposed in a glass-vial assay for 12 h. Mean mycosis rates followed same letter are not significantly different among the different concentrations of *Beauveria bassiana* ARP14 (small letters) or GHA (capital letters) (χ^2^, *P* > 0.05). Significance of differences between mycosis rates within conidial concentrations, between the ARP14 and GHA strains are denoted as follows: * 0.01<*P* ≤ 0.05, ** 0.001<*P* ≤ 0.01, ***P ≤ 0.001 and ns indicates non-significance.

The mycosis rates of *B*. *bassiana* strains ARP14 and GHA varied with *R*. *pedestris* life stage and showed concentration dependence. The mycosis rate for 2^nd^ instar *R*. *pedestris* nymphs was found to be significantly different among the concentrations of ARP14 (χ^2^_0.05_ *=* 109.03, *df =* 5, *P* < 0.001) and GHA (χ^2^_0.05_ *=* 89.54, *df =* 5, *P* < 0.001) (Fig 5). Significant effects of concentration were also observed in both 4^th^ instar nymphs and adult *R*. *pedestris* in ARP14 (4^th^ instar nymphs χ^2^_0.05_ *=* 137.89, *df =* 5, *P* < 0.001; adult females χ^2^_0.05_ *=* 49.99, *df =* 5, *P* < 0.001) and GHA strains (4^th^ instar nymph χ^2^_0.05_ *=* 113.17, *df =* 5, *P* < 0.001; adult females χ^2^_0.05_ *=* 39.43, *df =* 5, *P* < 0.001) (Fig 5).

The mycosis rates caused by the two fungal strains in 2^nd^ instar nymphs were similar (not significantly different) at three concentrations: 1×10^9^ (*Zc* = 1.15, *P* = 0.249); 1×10^8^ (*Zc* = 0.94, *P* = 0.349); and 1×10^5^ conidia/mL (*Zc* = 1.45, *P* = 0.147). However, ARP14 mycosis rates were 1.5 and 1.4 times higher than those caused by the GHA strain at 1×10^7^ (*Zc* = 2.28, *P* = 0.023) and 1×10^6^ conidia/mL (*Zc* = 2.00, *P* = 0.045), respectively (Fig 5). Similarly, the mycosis rates of 4^th^ instar nymphs caused by the two fungal strains were found to be similar for three concentrations: 1×10^9^ (*Zc* = 0.15, *P* = 0.879), 1×10^6^ (*Zc* = 1.35, *P* = 0.176), and 1×10^5^ conidia/mL (*Zc* = 1.60, *P* = 0.109).^.^ But for two concentrations, the mycosis rates differed between fungal strains: that of ARP14 was 1.3 times higher in both the 1×10^8^ (*Zc* = 3.08, *P* = 0.002) and the 1×10^7^ conidia/mL concentrations (*Zc* = 2.22, *P* = 0.026) (Fig 5). In adults, the mycosis rates caused by the two fungal strains were not significantly different for two concentrations: 1×10^9^ (*Zc* = 1.40, *P* = 0.161) and 1×10^5^ conidia/mL (*Zc* = 1.40, *P* = 0.161). But for three concentrations, the mycosis rates differed between fungal strains: ARP14 mycosis rate was 1.8, 2.0 and 2.5 times higher at 1×10^8^ (*Zc* = 2.26, *P* = 0.024), 1×10^7^ (*Zc* = 2.03, *P* = 0.043), and 1×10^6^ conidia/mL (*Zc* = 2.21, *P* = 0.027), respectively (Fig 5).

**Fig 6:**
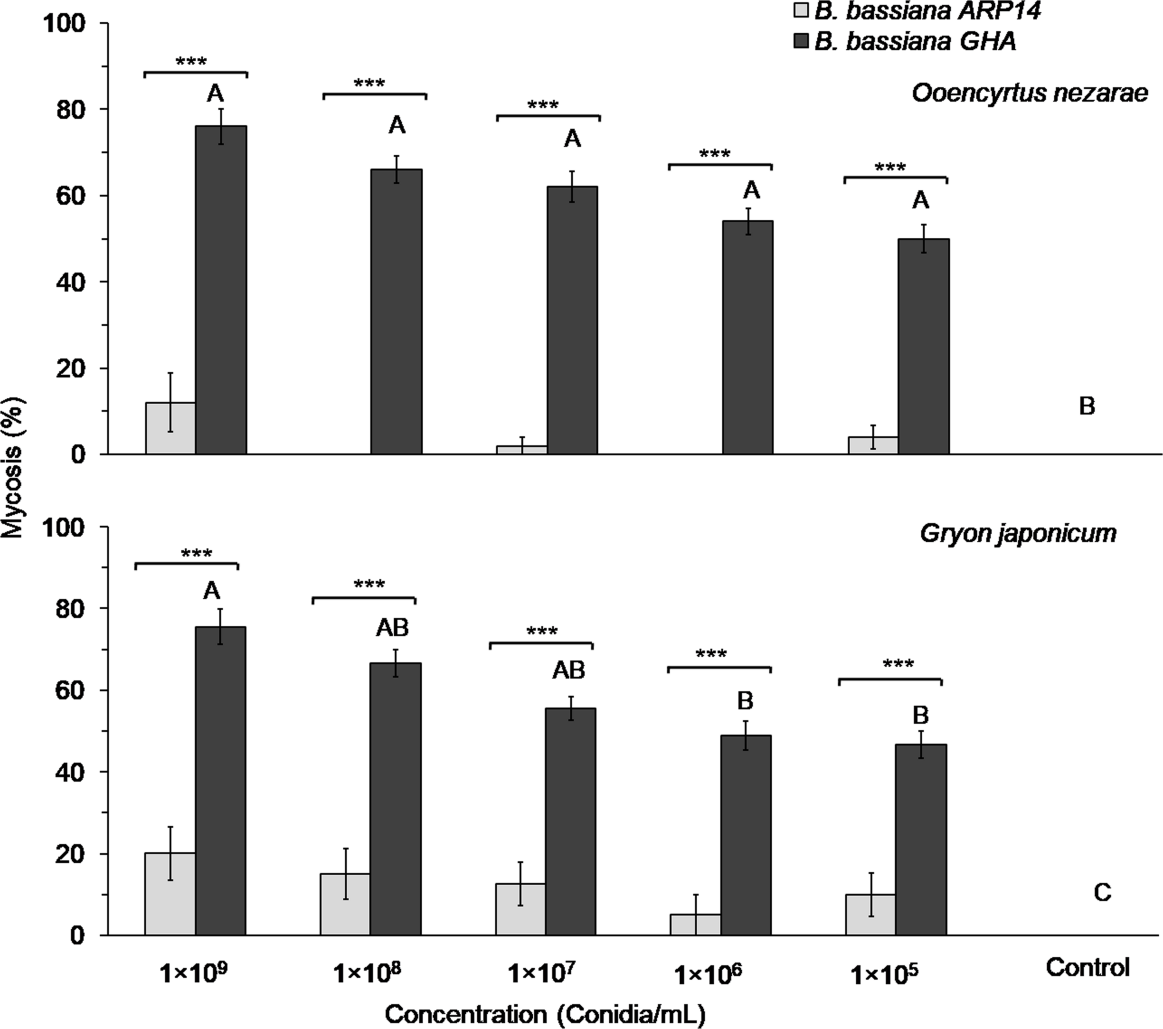
Mycosis rate of the *Beauveria bassiana* strains ARP14 and GHA in adults of *Ooencyrtus nezarae* and *Gryon japonicum* when exposed in conidia-coated glass-vials for 12 h. Mean mycosis rates followed same letter are not significantly different among the different concentrations in *Beauveria bassiana* ARP14 (small letters) or GHA (capital letters) (χ^2^, *P* > 0.05). Significance of differences between mycosis rates within conidial concentrations, between the ARP14 and GHA strains are denoted as follows: * 0.01<*P* ≤ 0.05, ** 0.001<*P* ≤ 0.01, ***P ≤ 0.001 and ns indicates non-significance.

The mycosis rates of *B*. *bassiana* GHA showed concentration dependence on *O*. *nezarae* and *G*. *japonicum*. Significant effect of concentrations were observed in *O*. *nezarae* (χ^2^_0.05_ *=* 71.68, *df =* 5, *P* < 0.001) and *G*. *japonicum* (χ^2^_0.05_ *=* 75.90, *df =* 5, *P* < 0.001) (Fig 6.) Interestingly, the mycosis development rate in *O*. *nezarae* caused by ARP14 was much lower than that caused by the GHA strain at all concentrations, i.e., 1×10^9^ (*Zc* = 6.45, *P* < 0.001), 1×10^8^ (*Zc* = 7.02, *P* < 0.001), 1×10^7^ (*Zc* = 6.43, *P* < 0.001), 1×10^6^ (*Zc* = 6.08, *P* < 0.001), and 1×10^5^ conidia/mL (*Zc* = 5.18, *P* < 0.001) (Fig 6). A similar pattern was found in *G*. *japonicum* at all concentrations, i.e., 1×10^9^ (*Zc* = 5.11, *P* < 0.001), 1×10^8^ (*Zc* = 4.81, *P* < 0.001), 1×10^7^ (*Zc* = 4.15, *P* < 0.001), 1×10^6^ (*Zc* = 4.48, *P* < 0.001), and 1× 10^5^ conidia/mL (*Zc* = 3.70, *P* < 0.001) (Fig 6).

## Discussion

The new entomopathogenic fungal isolate collected from *R*. *pedestris* was identified as *B*. *bassiana* and designated as strain ARP14, based on morphology [2, 34] and intraspecies and interspecies divergence rate with different *Beauveria* species and strains [35]. Strain ARP14 showed high virulence to *R*. *pedestris* in the glass-vial assay, and mortality rates of the tested life stages of *R*. *pedestris* increased with conidial concentration. The LC_50_ of strain ARP14 was not significantly different from that of GHA in any of the tested life stages of *R*. *pedestris*. Nevertheless, both ARP14 and GHA strains were found to be more effective against nymphal stages than the adult stage of *R*. *pedestris*. In a study conducted on *Riptortus linearis* (L.), *B*. *bassiana* CH1 was also more virulent to nymphal stages than adults [36]. A similar result was also found in other *B*. *bassiana* isolates, as for example where the larval stage of *Alphitobius diaperinus* (Panzer) (Coleoptera: Tenebrionidae) was more susceptible to *B*. *bassiana* than adults [37]. However, the LC_50_ of *B*. *bassiana* CPD9 strain in *Clavigralla tomentosicollis* Stål. (Hemiptera: Coreidae) was not different between 5^th^ instar nymphs and adults [38]. Similarly, other *B*. *bassiana* isolates/strains showed similar virulence to nymphs and adults of several hemipteran bugs [23,36,39,40]. The efficacy of EPF is known to vary, depending upon the host’s physiological state (i.e., weakened, ill, or low-immune condition) [41]. However, the mycosis rate of ARP14 in different life stages of *R*. *pedestris* was comparatively higher than the rates caused by the GHA strain, probably because ARP14 was isolated from *R*. *pedestris*. EPF are known to be more virulent on their natal host species than on novel species [40].

EPF, including *B*. *bassiana*, often have a wide physiological and ecological host ranges. Therefore, the development of an ecologically selective strain is needed for them to be an effective mycoinsecticide. In our study, *B*. *bassiana* ARP14 caused lower rates of mycosis in the pest’s two-egg parasitoids, *G*. *japonicum* and *O*. *nezarae*, and thus may be a selective mycoinsecticide for control of *R*. *pedestris*. Among commercial formulations of *B*. *bassiana*, Naturalis®-O is known to be relatively safe to the natural enemies of whiteflies, such as *Encarsia formosa* Gahan (Hymenoptera: Aphelinidae) and *Orius insidiosus* (Say) (Hemiptera: Anthocoridae), and *Phytoseiulus persimilis* Athias-Henriot (Mesostigmata: Phytoseiidae), while it lacks selectivity for the aphid parasitoid *Aphidius colemani* Viereck (Hymenoptera: Braconidae) [42]. Similarly, *Beauveria brongniartii* (Saccas) Petch when used to suppress larvae of *Melolontha melolontha* L. (Coleoptera: Scarabaeidae) in a forest habitat were less infectious to the natural enemies of these chafers [43]. Although the underlying mechanism of the selectivity of ARP14 against natural enemies is unknown, the virulence of EPF is known to vary interspecifically due to differences in toxin production, chemical composition of the host’s epicuticle, host cleaning behavior of the host (which removes conidia), and the method used to apply the conidia [41,42,44,45]. Exact effects of how such fungi may or may not differentially affect the target pest versus its natural enemies cannot be easily predicted, and studies are required in each system to determine if a product will have beneficial ecological selectivity.

In conclusion, as a mycoinsecticide with a low negative effect on key non-target egg parasitoids that could be used in a compatible manner with natural enemies in IPM [24], *B*. *bassiana* ARP14 appears to be a good candidate for use against *R*. *pedestris* while having minimal effect on the pest’s egg parasitoids. Nevertheless, development of formulation and verification of the efficacy in fields should be preceded before the application.

## Supporting information

S1 File Mortality and mycosis rate. (XLSX)
